# The Value of Quantitative Faecal Immunochemical Testing as a Prioritisation Tool for the Endoscopic Investigation of Patients With Iron Deficiency

**DOI:** 10.3389/fmed.2021.700753

**Published:** 2021-07-22

**Authors:** William Clackett, Stephen T. Barclay, Adrian J. Stanley, Aidan Cahill

**Affiliations:** Department of Gastroenterology, Glasgow Royal Infirmary, National Health Service Greater Glasgow and Clyde, Glasgow, United Kingdom

**Keywords:** quantitative faecal immunochemical test, qFit, endoscopy, iron deficiency, endoscopy prioritisation

## Abstract

Difficulty in providing endoscopy for patients with iron deficiency anaemia (IDA) during the COVID-19 pandemic has highlighted the requirement for a prioritisation tool. We aimed to test the validity of qFIT as a prioritisation tool in patients with iron deficiency and its ability to identify patients with advanced neoplastic lesions (ANLs). Data collected from patients referred with biochemically proven iron deficiency (ferritin ≤ 15 μg/L) and synchronous qFIT who underwent full gastrointestinal investigation within NHS Greater Glasgow and Clyde was analysed retrospectively. Patients who did not undergo full investigation, defined as gastroscopy and colonoscopy or CT colonography, were excluded. ANLs were defined as defined as upper GI cancer, colorectal adenoma ≥ 1 cm or colorectal cancer. Area under the curve (AUC) analysis was performed on qFIT results and outcome, defined as the presence of an ANL. AUC analysis guided cut-off scores for qFIT. Patients with a qFIT of <10, 10–200, >200, were allocated a score of 1, 2, and 3, respectively. A total of 575 patients met criteria for inclusion into the study. Overall, qFIT results strongly predicted the presence of ANLs (AUC 0.87, CI 0.81–0.92; *P* < 0.001). The prevalence of ANLs in patients with scores 1–3 was 1.2, 13.5, and 38.9% respectfully. When controlled for other significant variables, patients with a higher qFIT score were statistically more likely to have an ANL (qFIT score = 2; OR 12.8; *P* < 0.001, qFIT score = 3, OR 50.0; *P* < 0.001). A negative qFIT had a high NPV for the presence of ANLs (98.8%, CI 97.0–99.5%). These results strongly suggest that qFIT has validity as a prioritisation tool in patients with iron deficiency; both allowing for a more informed decision of investigation of patients with very low risk of malignancy, and in identifying higher risk patients who may benefit from more urgent endoscopy.

## Introduction

Iron deficiency anaemia (IDA) remains a prevalent condition affecting 2–5% of adult men and post-menopausal women in developed nations (1) and is a frequent cause for referral for endoscopic investigation (3–13%) in the UK (2). Guidelines produced by The British Society of Gastroenterology (BSG) suggest the use of bidirectional endoscopy to investigate these groups (3) and more recent guidance published by the American Gastroenterology Association also includes pre-menopausal women with asymptomatic IDA (4). Bidirectional endoscopy in post-menopausal women and men will reveal upper gastrointestinal (GI) malignancy in 2% and lower GI malignancy in 8.9% (5). The investigation of patients with iron deficiency without anaemia (IDWA) is not generally recommended by guidelines because it is thought that the overall risk of GI cancer is low in this patient group iron deficiency without anaemia (IDWA) (3).

In the context of a reduced endoscopy capacity and delivery of services as a result of the ongoing COVID-19 pandemic, providing timely bidirectional endoscopic investigations for patients with IDA is challenging. There is concern that limited endoscopy service provision as a result of the pandemic will result in delayed diagnosis of underlying GI neoplasia (6). A strategy to identify patients that may be at increased risk of significant GI pathology as a cause of IDA and who would benefit from earlier endoscopy is therefore necessary. We therefore aimed to test the validity of qFIT as an endoscopy prioritisation tool in patients with iron deficiency with or without anaemia that may facilitate appropriate triage during the current COVID pandemic, and aid risk stratification during the post-COVID recovery period and beyond.

## Patients/Materials And Methods

We searched laboratory databases for patients with proven iron deficiency across Greater Glasgow and Clyde health board between January 1, 2019 and December 31, 2019 and matched these with synchronous qFIT (HM-JACKarc) results. Patients were identified by interrogation of the TrakCare^®^ healthcare information system and information regarding patient demographics, referrals, clinical details, investigations and outcomes were recorded. Biochemically proven iron deficiency was defined as a ferritin ≤ 15 μg/L. Patients who did not undergo full investigation, defined as gastroscopy and colonoscopy or CT colonography, were excluded from the study. All patients had investigations carried out within 6 months of qFIT testing. Outcome from radiological or endoscopic investigation was obtained from electronic clinical notes. The presence of GI symptoms and recurrent anaemia was obtained from referral letters from primary care physicians and electronic clinical records. Recurrent anaemia was defined as the presence of anaemia more than 2 years prior to investigation. A positive qFIT was defined as ≥10 μg Hb/g. We defined advanced neoplastic lesion (ANL) as upper gastrointestinal cancer, colorectal adenoma ≥1 cm or colorectal cancer (CRC). We defined other significant lesions as gastric/duodenal ulceration, reflux oesophagitis (LA classification C/D), vascular abnormalities, polyp ≥1 cm and active inflammatory bowel disease.

All calculations were carried out using SPSS version 23 (Armonk, NY: IBM Corp) and R 4.0.0 for Mac (http://www.R-project.org). All results are expressed as the mean ± standard error for continuous variables, or as a percentage for categorical variables. Student *T*-test, Or Welch *T*-Test was carried out to compare the means of continuous parametric and non-parametric variables, respectively. Chi-squared tests were used for comparison of categorical variables. All tests were two-tailed and a value whereby *P* < 0.05 was considered as statistical significance.

Area under the receiver operator curve (AUROC) was conducted to determine the predictive validity of qFIT for detecting ANL. A cut-off of <10 μg Hb/g Hb/g was used as this currently is the cut-off used by NICE to determine if a test is negative. We then calculated the furthest co-ordinate from this which maintained an acceptable Youden Index; this corresponded to a cut-off of 200 μg Hb/g Hb/g. All qFIT results were grouped 1, 2, and 3 if a result fell into a range of <10 μg Hb/g Hb/g, 10–200, μg Hb/g and >200 μg Hb/g, respectively. Variables which displayed statistical significance at univariate analysis were considered for hierarchical analysis within the multivariate logistic regression equation. Regression was used to calculate odds ratios for qFIT scores and their association with ANLs. Positive predictive values (PPV), negative predictive values (NPV), sensitivity and specificity were calculated, with 95% confidence intervals (CI) for all categorised GI pathology.

## Results

A total of 1,769 patients had proven iron deficiency with a matched ferritin and qFIT result. Within this cohort, 944 patients received at least one type of endoscopic or radiological investigation and 575 patients underwent full GI investigation as described above for inclusion into the study. Descriptive statistics for common demographic and clinical parameters are recorded in (Table 1). A total of 50 patients had a confirmed ANL. All patients were iron deficient but almost 20% were not anaemic at referral, and this included seven patients with ANLs (four of whom had CRC). We found additional significant upper GI lesions in 41 patients (7.2%) and significant lower GI lesions in 20 patients (3.5%). All colorectal adenomas had evidence of low-grade dysplasia only. Mean qFIT for patients without ANLs were statistically lower than for patients with ANLs (55.6 ± 5.0 μg Hb/g vs. 208.7 ± 22.4 μg Hb/g; *P* < 0.001). There were two patients who had concurrent upper and lower gastrointestinal pathology at time of endoscopy: patient 1; gastric ulcer/ileal ulcer and patient 2; gastric ulcer/proctitis.

**Table 1:** Demographic and clinical parameters.

**Parameter**	**Total** **(*N* = 575)**	**No ANL** **(*N* = 525)**	**ANL** **(*N* = 50)**	***P*-value**
Age (years)	66.4 ± 1.09	65.9 ± 1.18	71.2 ± 1.70	0.172
Sex (% male)	34.3%	33.5%	42.0%	0.228
IDA (% present)	81.6%	81.1%	86.0%	0.397
Symptoms (% present)	46.4%	47.4%	36.0%	0.122
Recurrent anaemia (% present)	18.1%	23.5%	8%	0.052
Haemaglobin (g/L)	109.5 ± 0.67	109.7 ± 0.68	107.6 ± 2.95	0.494
Antiplatelet treatment (% present)	28.3%	29.1%	20.0%	0.170
Anticoagulant treatment (% present)	6.1%	5.1%	16.0%	0.002
qFIT (μg Hb/g)	55.6 ± 5.0	41.1 ± 3.90	208.7 ± 22.4	<0.001

Cut-off analysis aided the allocation of groupings by qFIT results of <10, 10–200, and >200 μg Hb/g. Table 2 outlines the subtype and frequency of neoplasm observed for each qFIT group. In patients with a qFIT <10, 10–200, >200 μg Hb/g we found ANL in 1.19, 13.5, and 38.9%, respectively. With these groupings we found cancer in 0.3, 8, and 28%, respectively.

**Table 2:** Frequency and subtype of advanced neoplastic lesion observed correlated with qFIT.

**qFIT group (μg Hb/g)**	**Cases** **(%)**	**No. ANL (%)**	**Type of ANL (N)**	**Cancer (%)**
<10	336 (58.4%)	4 (1.19%)	Colorectal adenoma (3)	0.3%
			Colorectal cancer (1)	
10–200	185 (32.2%)	25(13.5%)	Colorectal adenoma (10)	8%
			Colorectal cancer (11)	
			Oesophageal cancer (4)	
>200	54 (9.4%)	21 (38.9%)	Colorectal adenoma (6)	28%
			Colorectal cancer (14)	
			Oesophageal cancer (1)	

**Figure 1:**
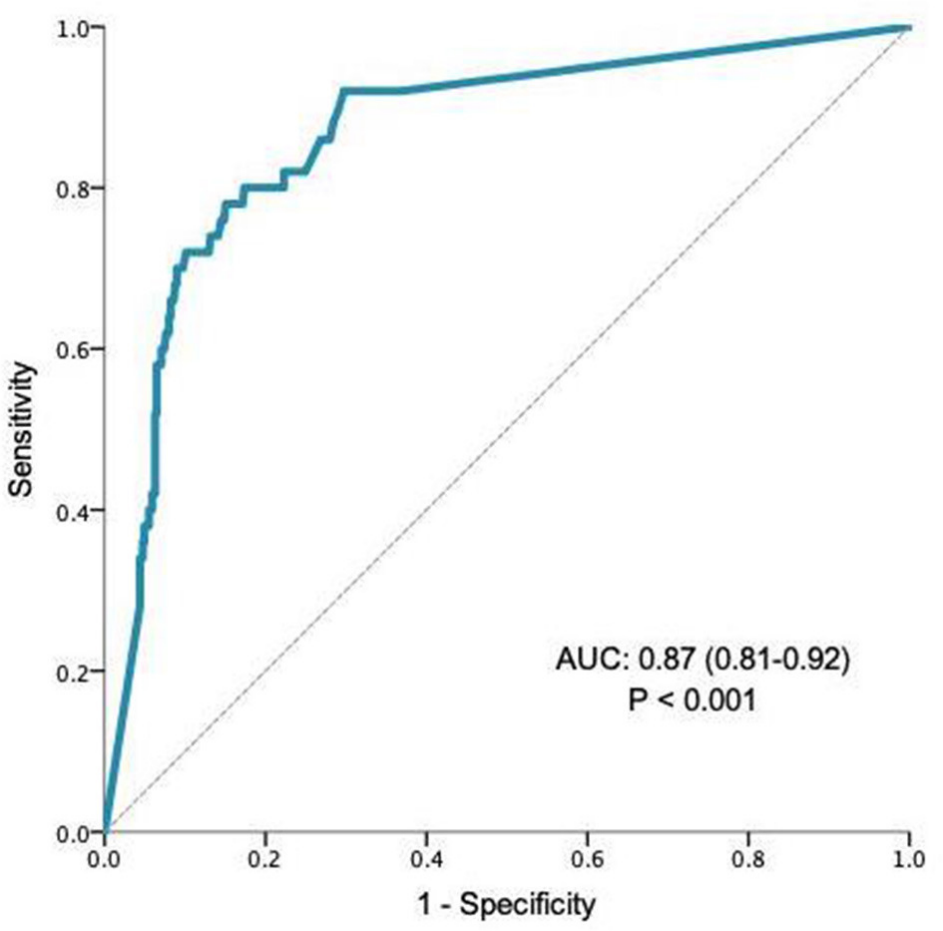
AUROC analysis for qFIT and accuracy in predicting ANL.

The validity of qFIT as a prioritisation tool was tested in AUROC analysis which showed that qFIT significantly predicted the presence of ANL (AUC 0.87, CI 0.81–0.92; *P* < 0.01, Figure 1). Moreover, qFIT was highly sensitive for the presence of ANL [92.0% (84.4–99.5%)], with moderate specificity [63.2% (59.1–67.4%)]. A negative qFIT had a high NPV for the presence of ANL (98.8% 97.0–99.5%). For the exclusion of cancer (as opposed to all neoplastic lesions) the NPV was 99.7% (98.0–99.9%) Performance characteristics for qFIT in relation to other GI findings are shown in Table 3.

**Table 3:** Diagnostic performance of qFIT for ANLs and additional significant GI pathology.

**Disease**	**Cases**	**Sensitivity**	**Specificity**	**PPV**	**NPV**
Gastric ulcer/severe oesophagitis	17 (3%)	35.3 (14.2–61.7)	49.1 (44.5–53.8)	2.51 (1.33–4.71)	96.7 (95.4–97.7)
Upper GI cancer	5 (0.9%)	100 (47.8–100)	59.0 (54.8–63.0)	2.09 (1.90–2.30)	100
Other upper GI pathology (GAVE, AVM, PHG, adenoma)	24 (4.2%)	50.0 (29.1–70.9)	58.8 (54.6–63.0)	5.02 (3.38–7.39)	96.4 (94.7–97.6)
Colorectal adenoma	19 (3.3%)	84.2 (60.4–96.6)	59.9 (55.7–64.0)	6.69 (5.45–8.20)	99.1 (97.51–99.7)
Colorectal cancer	26 (4.5%)	96.2 (80.4–99.9)	61.0 (56.8–65.1)	10.5 (9.30–11.7)	99.7 (98.0–100)
Other lower GI pathology (IBD, AVM)	20 (3.5%)	72.7 (49.8–89.3)	59.7 (55.5–63.8)	6.69 (5.17–8.63)	98.2 (96.5–99.1)

During univariate analysis only qFIT result and treatment with anticoagulation were associated with a higher prevalence of ANL. When these variables were analysed together with multivariate logistic regression (Table 4) the prevalence of ANL was significantly higher in patients with a qFIT 10–200 μg (OR 12.8, CI 4.4–37.4; *P* < 0.001) and higher still in patients with a qFIT >200 μg (OR 50.0, CI 16.0–156.3; *P* < 0.001). Following adjustment for qFIT group within the multivariate logistic regression the association between anticoagulation and presence of ANL was lost.

**Table 4:** Multivariate logistic regression analysis.

**Parameter**	**Odds ratio (displayed with CI)**	***P*-value**
Anticoagulation	2.13 (0.81–5.58)	0.16
Recurrent anaemia	0.32 (0.10–0.96)	0.04
qFIT <10 μg	-	-
qFIT 10–200 μg	12.8 (4.35–37.4)	<0.001
qFIT >200 μg	50.0 (16.0–156.3)	<0.001

Patients who were referred with recurrent anaemia had a non-significant lower risk of ANL (23.5 vs. 8%; *P* = 0.05). Though patients who were receiving anticoagulant therapy were more likely to be associated with higher rates of ANL (16 vs. 5.1%; *P* = 0.002), this was not observed in patients who were receiving antiplatelet therapy (20 vs. 29.1%; *P* = 0.170).

## Discussion

The Quantitative Faecal Immunochemical Test (qFIT) has been established as an accurate test in identifying colorectal cancer (CRC) and is used for national bowel cancer screening programs (7). Currently it is also used in the UK to guide referral in patients with colorectal symptoms who do not meet criteria for urgent suspicion of cancer (USOC) pathways (8, 9). A recent study supports the use of qFIT for symptomatic patients as a means to triage for urgency of colorectal referral during the COVID-19 recovery phase (10). There is limited published data about the use of qFIT to assist in the investigation of iron deficiency (11–14). One study suggests those with positive qFIT and IDA should be given a higher priority for colonoscopy over those with positive qFIT and no IDA (12).

The results from our study support a role for using qFIT to help prioritise the endoscopic investigation of patients referred with iron deficiency to gastroenterology out-patient services. The strong correlation with qFIT levels not only identifies those with an increased risk of having ANL dependent on the qFIT value but also confidently identifies those with a significantly lower risk.

We have suggested cut off values for qFIT results aided by AUROC analysis which offer a pragmatic risk stratification tool for clinicians who are investigating patients with iron deficiency. We found an ANL (cancer) in 1.19% (0.3%) of those with qFIT <10 μg, in 13.5% (8%) of those with qFIT 10–200 μg and in 38.9% (28%) of those with qFIT > 200 μg (Table 2). We have demonstrated that with increasing qFIT levels, there is an associated increase risk of underlying ANL and this is consistent with previous studies looking at patients with IDA and symptomatic colorectal referrals (10). We found an increasing risk of ANL being diagnosed in those with qFIT 10–200 μg (OR 12.8) and qFIT > 200 μg (OR 50.0) which may prove useful as a prioritisation tool for endoscopic investigation. To the best of our knowledge, this is the first study to use the aforementioned cut-offs in risk stratification of ANL in iron deficiency.

Our study has demonstrated that qFIT has an excellent NPV for identifying patients with iron deficiency who do not have ANL [98.8% (97.0–99.5%)]. In a pandemic, it may be more appropriate to use a test to exclude cancer, on the presumption that significant polyps will be picked up with resumption of bowel cancer screening programmes. Using this criteria, a qFIT would have a NPV of 99.7% in our population, and applying this cut off would have avoided 550/943 (58.3%) patients undergoing investigation. Although our results are in the broader context of iron deficiency, they are in keeping with previous studies evaluating the predictive validity of qFIT in patients with iron deficiency anaemia which also demonstrate a high NPV with a negative qFIT (11, 13, 14).

In addition, we included the diagnostic performance of qFIT for other significant GI pathology. We found other significant upper GI lesions in 7.2% and lower GI lesions in 3.5% of cases (Table 3). Overall, qFIT had a poorer sensitivity for detecting additional significant GI pathology, particularly those in the upper GI tract.

There is little information around the utility of qFIT in detecting upper GI malignancy. In our study, qFIT appeared to detect all patients with upper GI malignancy Importantly, however the sample size within this group is low and caution should be taken with its interpretation. Currently it is not recommended that FOB testing or qFIT be used as a diagnostic tool in the investigation of IDA (BSG guidelines), and its use in the assessment of upper GI blood loss and malignancy is controversial (15). This is largely reflective of the lack of evidence supporting the role of qFIT and prediction of upper GI malignancy, as well as the hypothesis that the destruction of haemoglobin in the upper GI tract by digestive peptidases may lead to poorer sensitivity (16). Our data adds to a small number of studies (17, 18) suggesting the further exploration of utilising qFIT in identifying upper GI malignancy. A recent study investigating the role of qFIT in rationalising endoscopy for patients with IDA has suggested carrying out gastroscopy independently of the qFIT result (13). We note however a recent prospective study in symptomatic patients found the risk of upper GI malignancy was independent of the qFIT result (19). Further research will be required to identify if qFIT can also assist in rationalising referrals for upper GI malignancy.

In our group of patients age, sex and haemoglobin values were not independent risk factors for underlying ANLs as has been suggested in previous studies (20). It is important to note that in our study we included patients with iron deficiency though not anaemia. This proved to be clinically justified as we found a proportion of patients with ANL (14%) who had biochemically proven iron deficiency but were not anaemic at time of referral. A recent meta-analysis by Alexandre et al. has suggested that older patients (>50 years) and non-screened populations are at higher risk and require endoscopic investigation. It has been observed that although the overall prevalence of CRC in IDWA is low, there is an elevated risk of CRC within these groups with IDWA (21). Exclusion of patients with IDWA may result in a cohort of ANL being missed or presenting later in the disease course. Further research is required to clarify the role of qFIT testing in the investigation of patients with IDWA.

Within this study, we observed a non-significant trend between a lower prevalence of ANL and the history of recurrent anaemia. These patients were noted to have been iron deficient more than 2 years prior to current referral episode. A small but significant yield in follow up or following repeat bidirectional endoscopy has previously been reported (22, 23). We speculate that the lower yield in this group of patients is either because some of these patients will have had previous negative endoscopic investigation or because of the longer lag time involved and are therefore less likely to have significant underlying pathology. This is an area lacking in evidence that warrants further evaluation on the utility of repeating tests in those with persistent or recurrent iron deficiency with prior negative investigations as was noted in the recently published AGA guidance (4).

Almost half of our study population were recorded as being symptomatic at referral or at time of investigation, however we observed that the presence of GI symptoms and iron deficiency was not associated with increased finding of ANL. Although it was out with the scope of this paper to test the significance of individual symptoms and their association with neoplasia we note that previous meta-analyses assessing the predictive value of individual symptoms have had inconsistent results (24–26).

We recognise that our study had a number of limitations. Firstly, it was a retrospective study of clinical records of patients referred over a 12 month period. Because it is retrospective, we therefore could not account for all potential bias. A degree of selection bias is likely due to the fact that patients with IDWA are not generally referred for bidirectional endoscopy unless there are other factors influencing the decision for referral. There was a relatively short follow-up period. All patients with a documented ferritin ≤ 15 μg/L during this period were not included as they did not have matched qFIT results available. Approximately 50% of patients with matched qFIT results were either not referred for investigation or were pending investigation at the time of data collection. Data collected regarding patient symptoms were gathered from primary care referral letters, clinical letters and endoscopy reports which may have varying degrees of accuracy. Moreover, we did not collect data on the location of colorectal adenomas that were observed at endoscopy. Although all investigations were completed within 6 months, we have not specified the exact time from qFIT test to investigation, possibly not observing an interaction between delay to investigation and diagnosis of ANL. A final limitation is that data on patients with small bowel lesions was not collected; we therefore cannot comment on the utility of qFIT for this patient group. However, we note current guidance (3) does not recommend routine investigation of the small bowel in IDA.

In conclusion, our study strongly supports the role of qFIT as a prioritisation tool in patients with iron deficiency. During the current global pandemic endoscopy services may benefit from being able to prioritise the investigation of patients based on the qFIT level to expedite investigation of those at highest risk. For those with a negative qFIT, investigation may take place at a lower priority, or in the context of a very low likelihood of significant pathology, allow a better informed discussion with patients around the likely benefit of undergoing investigation. Whilst such an approach is of clear benefit during time of limitation of endoscopic resources, it also likely to be beneficial in the recovery period and beyond.

## Data Availability Statement

The raw data supporting the conclusions of this article will be made available by the authors, without undue reservation.

## Author Contributions

WC responsible for data analysis, literature review, and article write up. AC responsible for project design and data collection. SB and AS contributors to data analysis and literature review. All authors contributed to the article and approved the submitted version.

## Conflict of Interest

The authors declare that the research was conducted in the absence of any commercial or financial relationships that could be construed as a potential conflict of interest.
